# Can a pill prevent HIV? Negotiating the biomedicalisation of HIV prevention

**DOI:** 10.1111/1467-9566.12372

**Published:** 2015-10-26

**Authors:** Ingrid Young, Paul Flowers, Lisa McDaid

**Affiliations:** ^1^ MRC/CSO Social and Public Health Sciences UnitUniversity of GlasgowUK; ^2^ School of Health and Life SciencesGlasgow Caledonian UniversityUK

**Keywords:** HIV, sexual health, focus groups, biomedical, gay/lesbian, immigrants/migrants/refugees

## Abstract

This article examines how biomedicalisation is encountered, responded to and negotiated within and in relation to new biomedical forms of HIV prevention. We draw on exploratory focus group discussions on pre‐exposure prophylaxis (PrEP) and treatment as prevention (TasP) to examine how the processes of biomedicalisation are affected by and affect the diverse experiences of communities who have been epidemiologically framed as ‘vulnerable’ to HIV and towards whom PrEP and TasP will most likely be targeted. We found that participants were largely critical of the perceived commodification of HIV prevention as seen through PrEP, although this was in tension with the construction of being medical consumers by potential PrEP candidates. We also found how deeply entrenched forms of HIV stigma and homophobia can shape and obfuscate the consumption and management of HIV‐related knowledge. Finally, we found that rather than seeing TasP or PrEP as ‘liberating’ through reduced levels of infectiousness or risk of transmission, social and legal requirements of responsibility in relation to HIV risk reinforced unequal forms of biomedical self‐governance. Overall, we found that the stratifying processes of biomedicalisation will have significant implications in how TasP, PrEP and HIV prevention more generally are negotiated.

## Introduction

Since 2010, a number of major international trials have demonstrated the efficacy of using antiretrovirals (ARVs) to prevent the transmission of HIV (Cohen *et al*. 2012, Baeten and Grant 2013). Unlike research for an HIV vaccine or cure, which seeks to develop new drugs to prevent acquisition of or eradicate the virus, these trials demonstrated the potential of existing pharmaceuticals to prevent and manage the sexual transmission of HIV in two new ways. Treatment as prevention (TasP) is the use of existing treatment to reduce levels of infectiousness in people living with HIV. Pre‐exposure prophylaxis (PrEP) is the use of ARVs by people who are HIV‐negative to prevent acquisition of the virus. Together, these two interventions have garnered significant international attention and there have been calls to make them available to those most at risk of HIV where they are not already available (Jones 2014, *The Lancet HIV* 2014). Nevertheless, such interventions have also attracted much criticism. Kippax and colleagues (Nguyen *et al*. 2011, Kippax and Stephenson 2012) have argued that these new interventions are not a panacea for HIV prevention, but need to be part of a broader approach which integrates social and behavioural dimensions and contexts alongside these biomedical interventions. Moreover, Kippax has argued that this apparent shift away from behavioural (i.e. condom‐based strategies) to biomedical interventions in the form of PrEP and TasP is not new but situated in the growing biomedicalisation of HIV prevention (Kippax and Race 2003, Kippax and Stephenson 2012). These authors trace the shift from social to biomedical narratives of HIV prevention, attributing the discovery of effective HIV treatment in 1996 as the pivotal moment in which the biomedical narrative – and approach – really takes hold. The consequence, Kippax and Stephenson (2012: 796) explain, is that ‘increasing biomedicalisation is distorting prevention efforts’. Instead, they argue for a ‘social public health’ approach to HIV prevention, which includes biomedical prevention interventions, but which also engages with how HIV is encountered ‘in life – as biological, material, as informational and technological, as emotional and affective, as social, collective, institutional’(Kippax and Stephenson 2012: 796).

Other have traced the technological shift towards the biomedical much earlier, with the introduction of the HIV antibody test in the mid‐1980s and the emergence of a new biomedical identity based on sero‐status (Flowers 2001, Race 2001). This corresponds with what Clarke *et al*. (2003) describe as a wider shift towards biomedicalisation across an expanding number of health – and increasingly non‐health – arenas. From approximately 1985, these authors chart a significant shift from medicalisation to biomedicalisation. That is, there has been a move from ‘medicine exerting clinical and social *control over* particular conditions to an increasingly techno‐scientifically constituted biomedicine also capable of effecting the *transformation of* bodies and lives’ (Clarke *et al*. 2003: 165). However, rather than viewing biomedicalisation as deterministic, they see human action and technoscience as ‘co‐constitutive’. Their project in charting biomedicalisation is to identify and create new critical spaces to enable ‘greater democratic participation in shaping human futures *with* technosciences’ (Clarke *et al*. 2003: 166). This article responds to Clarke *et al*.'s project and examines how biomedicalisation is encountered, responded to and negotiated within and in relation to new biomedical forms of HIV prevention. We draw on exploratory focus group discussions on PrEP and TasP with participants from diverse communities affected by and/or at risk of HIV in the UK. We consider how the processes of biomedicalisation are affected by and affect the diverse experiences of communities who have been epidemiologically framed as ‘vulnerable’ to HIV and towards whom PrEP and TasP will most likely be targeted in public health policy. In particular, we consider how PrEP and TasP are imagined, produced and negotiated through the diverse social, material and technological environments in which study participants inhabit, and within a broader framework of the biomedicalisation of HIV prevention.

## Biomedicalisation

Where Kippax and Stephenson note the impact of biomedicalisation on the specific direction of HIV prevention research, policy and practice, the broader biomedicalisation thesis offers an overarching conceptual framework within which to understand many developments in contemporary health‐care (Clarke *et al*. 2010). Biomedicalisation addresses the complex networks and agents that constitute, and are constituted by novel, and often highly technologically mediated, processes of medicalisation. The broad and inclusive framework captures the fluid, technological and expanding repertoire of processes, structures and disciplines now appropriated into the exercise of what could be called biopower (Foucault 1984), and what has been described as the production of the biosocial (Rabinow 1996). Together these approaches stress the reciprocal interplay of the psychosocial, sociocultural, politico‐economic and biomedical domains. Clarke *et al*. (2010) detail five central tenets of this historical shift from medicalisation to biomedicalisation. First, major politico‐economic shifts enable expansion of the privatisation and commodification of medicine and medical research, with the resultant centralisation, rationalisation and devolution of health services. Second, there is a distinct focus upon health rather than illness per se, with a concomitant increase in new forms of risk and surveillance. Health is cast as a moral obligation as the body becomes a legitimate site for increased intervention, enabled and constituted through biomedical forms of self‐governance, enacted through health and biological citizenship (Petersen and Lupton 1996, Petryna 2002, Rose 2007). Third, the technological and scientific ways in which we understand health are expanding, based on: an increasing computerisation and centralised storage and organisation of medical data; a molecularisation and geneticisation of health (Rose 2007); and the transformation of medical developments through digitisation and hybridisation to create new biomedical technologies. Fourth, there is a transformation of information and the production and distribution of knowledge. This includes: an increase and diversification of health‐related information which challenges the lay‐expert knowledge divide; a co‐option of competing knowledge systems; and new techniques to legitimate biomedical claims (e.g. randomised control trials). Fifth, biomedicalisation results in the transformation of bodies and identities, ascribing new technoscientific and biosocial identities on both an individual and collective level (Rabinow 1996).

Biomedicalisation suggests that technoscience and biomedicine leach into, and construct, everyday life with a proliferation of risk and uncertainty. Biomedical research and its therapeutic applications, such as assisted reproductive technologies, genetic testing for cancer, and blood, organ and tissue transplantation, can be understood as playing an increasingly important role in the everyday experiences of health and illness (Petryna 2002, Waldby and Mitchell 2006, Lock and Nguyen 2010). However, the processes of biomedicalisation are both temporally uneven and stratified, with the potential to augment existing – and create new – forms of difference and health inequalities. And yet, biomedicalisation is not a ‘technoscientific tsunami that will obliterate prior practices and cultures’ (Clarke *et al*. 2003: 185) , but one which creates new sites of negotiation, knowledge, agency and citizenship. The heterogeneity of biomedicalisation and the co‐constitutive impact of different lived experiences necessitate focused examination of particular biomedicalisation processes, situated in their specific – but globalised – contexts. Developments in the preventative potential of HIV treatments through PrEP and TasP, legitimated by international clinical trials and expanding the technoscientific nature of HIV prevention practices, provide a productive arena in which to examine the emergence of biomedicalisation in a contemporary context.

## TasP and PrEP

In 2011, the clinical trial HPTN052 was stopped early when it determined that, when people living with HIV took ARVs earlier than the standard of care clinical indications, the risk of HIV transmission to sexual partners was significantly reduced. Confirming what had earlier been claimed in the *Swiss Statement* (Vernazza *et al*. 2008) but which was widely rejected due to lack of clinical trial evidence, HPTN052 trial findings demonstrated that ARVs used to manage HIV and lower HIV viral loads to ‘undetectable’ resulted in significantly reduced levels of infectiousness and reduced risk of transmission by 96 per cent (Cohen *et al*. 2012). Many public health researchers and policymakers responded to TasP by modelling, testing and measuring the wider public health benefits of early HIV treatment initiation across communities (HIV Modelling Consortium Treatment as Prevention Editorial Writing Group 2012). There is currently mixed clinical evidence as to the added health benefits of early treatment for individuals living with HIV, in spite of the global policy context which increasingly favours a TasP‐based approach to HIV prevention (WHO 2013). Moreover, these clinical findings have been translated into public health policy in diverse ways. For instance, the British HIV Association (BHIVA) has accepted the clinical evidence of TasP, but advises that starting treatment ‘early’ to prevent transmission to a sexual partner be explored on an individual patient basis. These guidelines add that any treatment decision must not be through undue pressure from a sexual partner and/or clinician (BHIVA and EAGA 2013). In contrast, the province of British Columbia (Canada) has implemented TasP policy which means that anyone diagnosed with HIV is to start treatment immediately (British Columbia Ministry of Health 2012). As increasingly more countries are exploring and/or implementing new treatment guidelines (Morlat *et al*. 2013), TasP is quickly becoming standard practice in HIV treatment and prevention decisions.

There have been many international, multi‐site clinical research trials funded and initiated to determine if PrEP was safe and effective as an HIV prevention method. These trials found daily and consistent use of ARVs would reduce the risk of HIV transmission, ranging between 44 per cent in the iPrEX trial in 2010, up to 86 per cent most recently in the PROUD trial with gay and bisexual men in the UK (Baeten and Grant 2013, McCormack 2015). Many have described drug adherence as critical to the clinical findings (Bangsberg 2014), with poor results of some studies attributed to lack of participant engagement with the trial itself, resulting in participants not taking the medication (van der Straten *et al*. 2014). In 2012, the US Food and Drug administration approved Truvada for use as PrEP (Food and Drug Administration (FDA) 2012), although the decision was not without controversy. Unlike TasP, public health policymakers and practitioners in many countries have so far been wary of officially sanctioning PrEP as an HIV prevention option on the grounds of mixed clinical evidence (McCormack *et al*. 2012), not to mention the potential for ‘mis‐use’. However, there is considerable activism and discussion aimed at widening access to PrEP beyond the US context, especially given recent clinical trials results in the UK (Jones 2014, The Lancet HIV 2014).

The potential of pharmaceutically‐based HIV prevention options has resulted in an uneven flurry of ‘acceptability’ research, which explores if and how potential PrEP or TasP ‘candidates’ might use these interventions. Much of this research has been framed largely through a fear of the potential abandonment of condoms (resulting in potentially increased risk of HIV transmission) and or that individuals will fail to take their medication regularly or consistently (Young and McDaid 2014). This narrow conceptualisation of ‘acceptability’ excludes a range of factors and contexts which might affect potential uptake and use of these interventions. Nevertheless some researchers have endeavoured to understand the wider social context in which PrEP or TasP might be used (Guest *et al*. 2010, Ware *et al*. 2012), or indeed, have sought to situate these relatively new interventions in a broader theoretical context (Rosengarten 2010, Persson 2013, Holt 2014). Most pertinent to our research, Persson (2013) highlights the need to better understand the potential dissonances between the biomedical rhetoric around emerging new biotechnological interventions and the lived experiences of those who might use, shape and be shaped by these technologies. In the remainder of this article, we will explore these potential dissonances through qualitative research conducted in Scotland.

## The study

Gay, bisexual and/or men who have sex with men^1^, and men and women from migrant African communities^2^ are disproportionately affected by HIV and currently represent over half of all new HIV diagnoses in Scotland (Health Protection Scotland 2013). Although these two communities are distinct from each other in terms of epidemiological, social and economic experiences of HIV (while also recognising the heterogeneity within these broad groupings), they represent the groups towards which PrEP and/or TasP will most likely be initially targeted in public health policy. HIV and the Biomedical is a qualitative study which explored how PrEP and/or TasP might be responded to and/or incorporated into the everyday lives of gay men, and African men and women living in Scotland. Drawing on well‐established qualitative methods (Barbour and Kitzinger 1999, Silverman 2000), we conducted exploratory focus groups (FGs) to explore and situate PrEP and TasP in a wider social context, paying particular attention to language, social dynamics and resonances/dissonances with sexual practice and other health technologies. This research informed subsequent in‐depth interviews, reported on elsewhere (Young *et al*. 2014). This article reports on the FGs themselves.

Participants were recruited through existing community and/or support groups in order to build on established group rapport and dynamics, enabling discussions of sensitive topics. Groups were identified with the assistance of community research partner sexual health and/or LGBT organisations based in Scotland who support regular groups to discuss HIV, sexual health and other support needs. We held the FGs in four cities reflecting a range of urban and semi‐rural Scottish locations, including its two largest cities. We conducted FGs with 33 participants who were either gay men or African men and women between August and November 2012. FGs were organised by sero‐status and ethnic and/or sexual identity. We ran five FGs with twenty‐two gay men, including three with HIV‐positive men, and two with HIV‐negative or untested men. We ran two FGs with 11 African men and women (eight women, three men), including one HIV‐positive group. Participants ranged in age from 18 to 75 years across all groups.

FG discussions were facilitated by IY and/or PF. Participants were first asked about their understandings and management of sexual health risks, paying particular attention to the role of sexual health technologies within these strategies. Participants were presented with a range of items, such as condoms (male and female), sachets of lubricant, a home pregnancy test, an emptied bottle of Truvada, a mock‐up bottle of antibiotics, a chart of available ARVs, images of Oraquick^®^ In‐Home HIV test and a rapid fingerprick HIV test. PrEP and TasP were then explained to participants with the help of visual aids (Young *et al*. 2014). Specific rates of effectiveness were not provided, but the facilitator explained the varying rates of efficacy identified in a number of clinical trials. Participants were then asked to discuss PrEP and TasP in relation to their own sexual health, including if and how they might be used. The study received ethical approval from the University of Glasgow College of Social Sciences Ethics Committee. Participants provided written consent before the discussion started and received £15 voucher and travel costs. FGs were digitally recorded, transcribed verbatim and names and any identifying features were removed from the transcriptions. Data were analysed thematically; IY and PF independently analysed half of the transcripts by identifying emerging themes and issues and made notes in an analysis booklet through the process of reading and rereading until saturation. Notes were guided by four analytic categories: descriptive/content; interactional dynamics; microanalysis/key discourse; and general theme. The analysed transcripts were then exchanged and re‐analysed, building on the original researcher's notes. Once each transcript had been seen and analysed twice, key themes were discussed and agreed. Rigour throughout the analysis was achieved through an iterative process of discussion and revision of findings (Barbour and Kitzinger 1999, Mason 1996, Silverman 2000). Here, we present three of the main themes to emerge from this analysis, which illustrate how the processes of biomedicalisation are negotiated and contested in relation to PrEP and TasP.

## Contesting the commodification of HIV prevention

Commodification and privatisation of medicine, the first tenant of biomedicalisation, shaped the responses of many participants. For instance, questioning the role that private, for‐profit pharmaceutical companies might have played in developing and marketing PrEP was a common response amongst HIV‐positive participants, and especially amongst HIV‐positive gay men. These critiques often shaped the questioning or even immediate rejection of PrEP as an acceptable prevention option. The perceived association between PrEP and ‘Big Pharma’ for many rendered these new pharmaceutically‐based interventions immediately suspect; participants suggested that the motivation for profits led them to question the scientific credibility and effectiveness of PrEP to actually work:

Respondent 1: How much does the tablet cost?

 

R2 It's a money making exercise for the Americans. That's all it is.

R3 It's like a multi‐vitamin. It doesnae [doesn't] work.

R4 Or is it a case of …

R2 No Americans, drug companies.

R3 Aye, totally, as well.

R2 It's the drug company's way of making money.

(HIV‐positive gay men)

The critique of PrEP as driven by profit was augmented by the claim that it was driven by American drug companies. Rather than seeing drug companies as part of multi‐national conglomerates, a characteristic of many contemporary depictions of pharmaceutical and globalization (Petryna 2009), their scepticism was grounded in a perception of the dominance of American companies in global medical markets. In contrast to a privatised, American health system epitomised by these companies, a free public health system funded through collective taxation appeared to be highly valued by HIV‐positive participants, most of whom accessed their own HIV treatment without direct cost to themselves. While the initial question in the above extract about cost of PrEP was overtaken by a collective rejection of American drug companies, it was an issue which emerged across many of the discussions in relation to PrEP provision within a publically funded health system. Conscious of the cost of their own medication, participants questioned the wisdom of creating more demand for expensive drugs in an already over‐stretched system:

I mean it costs a blooming fortune just to feed the existing people who are infected with these drugs. So to actually encourage the whole population to conduct themselves in such a way that they not only used protected facilities like condoms but then they take a wee [small] pill and we all remember what it was like when you started taking pills, they make you sick. So if the pill makes you sick, when you're healthy, it then, then you're gonna stop taking the pill. (HIV‐positive gay man)

Participants drew on their own experiences of starting treatment and enduring difficult and long‐lasting side effects to understand how PrEP might work for others and were concerned about the potential for financial waste if people stopped taking the drugs. By casting PrEP as optional and HIV‐negative PrEP users as healthy, HIV‐positive participants not only invoked a clear division between essential and non‐essential use of ARVs, but also the necessity of continued adherence. Given the potential risk of non‐adherence, availability of condoms and a finite set of resources, many HIV‐positive gay men deemed PrEP as inappropriate for a publicly funded health system. This not only speaks to the rationalisation of health services (Clarke *et al*. 2003: 170), but also to the economisation of health services which demands cost‐containment and compliant users (Ewert 2009).

HIV‐positive African participants also expressed concerns about the use of ARVs for prevention situated within the context of resource‐limited health systems. However, in contrast to the descriptions above, these participants drew on their experiences of HIV treatment and care outside of the UK to frame their understandings of PrEP and TasP. African participants came from a diverse range of countries, reflecting a mix of health system failures and successes in providing HIV treatment and care. In addition, FG participants reflected a wide range of experiences within the UK, ranging from recent arrivals to long‐term UK residency. Yet, within these diverse contexts, many of the HIV‐positive African participants described either family members’ or their own difficulties in accessing treatment, or having to rely on earlier generations of ARVs resulting in increased drug toxicity and tablet numbers. When one participant described taking nine tablets a day, others thought it was ‘too much’ in comparison to currently the standard of care in the UK (HIV‐positive African group). Grounded in this perceived unequal access to and burden of treatment, some participants critiqued the use of ARVs where they were not intended for people diagnosed with HIV, and even to criticise the wider language of HIV prevention and cure claims. One man explained:

We want to send a message to the scientists. Look when you talk about the guy who was cured in Germany seems they'll be looking at the rich not to the poor. Because that's not fair. Why they have to write something or call this a cure if they know there's billions of people cannot afford it? (HIV‐positive African man)

For this participant, the response to PrEP and TasP was embedded in the knowledge and experience of the global politics of biomedical research and discovery; participants were engaged in these politics in as much as they were aware of and directly affected by inequalities in access not only to new therapies, but to essential HIV treatments. This reaction highlights the way in which experiences of migration and global health inequities shape responses to PrEP and TasP within the context of the global markets of biomedical HIV prevention and care.

However, the critique of PrEP as an inappropriately commodified HIV prevention option was not shared amongst all participants. In particular, HIV‐negative and untested gay men expressed none of the same concerns about the role of American drug companies in the introduction of PrEP. While not unanimously embracing PrEP as a prevention option, these participants were concerned about more local (UK) inequalities of access:

R1 Like if they know it works why can they not bring it tae [to] Britain? If it's already been proved in America to be sold in America? Why not bring it to Britain? … ‘cause we're that bloomin’ much up each other's bums anyways …

R2 Is it not because … there's different laws in Europe than there is from America about what medication you're allowed tae [to] take an’ stuff … Like there's some in America that they're not allowed to have but we're allowed to have it over here, an’ there's ones that we can have over – like ones that we can't have over here that they're allowed over there … There's like rules an’ stuff of certain – like a certain –

R3 Totally, laws are different.

R1 See the folk in Parliament if they look at the statistics, if they reckon HIV's goin’ up an’ all that stuff, then – an’ if it's been proved in America – then they should just get off their high‐horses an’ change the law themselves.

R3 They'll have to sell it to us first ‘cause they've got it an’ they've produced it. They'll have to sell it to us won't they? If we wanted it in the UK, they'd have to sell it to us.

(HIV‐negative gay men)

In contrast to the criticisms by HIV‐positive participants, these men viewed the complexities of international drug markets as potentially obstructing access to PrEP. Indeed, seeing the government and international trade laws as the barrier to open markets for HIV prevention commodities signals the men's constructions of the medical consumer who should have access to these products should they choose. They explain that this is especially critical given the rising rates of HIV and the availability of an apparently effective prevention option. For these participants, it is the pharmaceutical market and not the national health system which enables access to critical HIV prevention solutions.

Although participants’ responses to PrEP are grounded in very different interpretations of the commodification of HIV prevention, they are all equally grounded in notions of equity in access. While those participants who are reliant on publically‐funded health systems for their care are concerned about non‐essential use of resources in shrinking health systems and potentially increasing inequalities in global markets, participants with limited engagement with health services to date view access and choice as critical factors in maintaining good health. What is important to note here is how the commodification of HIV prevention technologies and the notion of health equity shape the response to and acceptability of PrEP and whether (or not) it should be made available through existing, publically‐funded health infrastructures.

## Contesting stigma and access to knowledge

Clarke *et al*. (2003: 177) argue that the production and transmission of knowledge are key sites of biomedicalisation in relation to who is ‘responsible for grasping and applying such knowledges’. We found that participants were highly attuned to existing forms of HIV stigma, and its implications for access to PrEP, TasP or general biomedical HIV‐related information. Integral to discussions about TasP were anxieties about who would have access to this information because of the role it might play in reducing HIV‐related stigma. HIV‐positive gay men were concerned that this information was not available or understandable through existing, gay‐scene based messages for those not affected by HIV:

But if you're on tablets, it reduces the infectiousness of HIV if you've got an undetectable viral load so you're less risk to your partner, passing it on, and I think it's only people that know about HIV that really know about that, or people who've actually read into it or who've got friends who know about it as well. So it's all about word of mouth. That kind of thing's not advertised on the scene. There's so many sexual health messages out there as well.

(HIV‐positive gay man)

Participants were concerned that the existing sero‐divide (division on basis of HIV‐status) would negatively affect how TasP was understood: it was not the type of message available, nor would it filter through the myriad of sexual health messages currently in circulation. In the above extract, participants cite the importance of proximity to HIV (Keogh 2008) in accessing this information. Implicit in these observations is the notion that not living with HIV – or being close to those affected by HIV – shapes what information is accessible and who has access. Participants were concerned that existing forms of HIV‐stigma would preclude those not affected by HIV from engaging in this critical ‘new’ information about HIV risk.

Discussions highlighted how risk of HIV‐related stigma is often framed as ‘lacking knowledge’ and, as such, represents a complex challenge to the acceptability of PrEP/TasP. The following exchange with HIV‐negative African participants addresses the potential reaction to PrEP by others, and how this might be managed within the context of HIV‐related stigma that risks conferring an HIV‐positive identity to the potential PrEP user:

R1 The normal, normal to day‐to‐day person they think that, I mean it's that thing when people use medication to confirm somebody's illness it's almost like, ‘oh I saw that bottle, I saw that medicine’, or ‘they are on antibiotics that means that they are on that, that they've got that infection’ … It's almost like medication is … confirms people's suspicions.

R2 Yeah, but I think for this one it might be different because people would know what it does, you know they will know that some people can take it although they don't have the disease.

R1 How many, how many know that? You know? … We've got a true advantage, we know. Like me, before I can carry this out, I will know that everybody they are aware of it, like maybe through campaign or advertisement that this thing can cure it can help you the risk. Then, I can be able to take it along with me and when they ask me I will give it to them to read.

R3 If you had one partner, what would it mean for your partner? But would you not be, in a way, exposing your partner then if you are going around saying, ‘no I'm taking this medication’.

R1 No, no, no, you don't understand. It's not that. I'd be taking it like, like it might be in my, in my bag. You know maybe someone want to take something my bag, go an check it in my bag and saw it there.

R2 Yes. Well then you would explain to them –

R1 No. Only if, only if, that person asked, then I would give it to her to read, or to that person to read. Is for prevention. If it say it work? I say I dunno it's for prevention, to prevent myself.

(HIV‐Negative/untested African Participants)

In this extract, participants describe a shared understanding of HIV‐related stigma amongst their families and friends. One participant in particular describes a set of strategies to manage this stigma, which rely on distancing herself from HIV and limiting the possibilities of exposure to this stigma. Critical to this management is her own knowledge of PrEP and ability to communicate this, indicating the way in which PrEP users might be assumed to also need to be PrEP advocates. Moreover, she assumes that PrEP would be accompanied by an institutionally sanctioned education campaign, thus corroborating her story and decreasing the chances of her being ‘mistaken’ as HIV‐Positive. The expectation that biomedical knowledge about PrEP will be deployed in a very particular way points to the how shared perceptions of stigma interact with assumptions about who has access to PrEP knowledge.

Not only did HIV‐stigma play a significant role in the acceptability of PrEP and TasP, but it was an important factor in how HIV‐negative participants managed their own HIV‐negative identities and influenced discussions around HIV‐prevention more generally. One group of young, HIV‐negative/untested gay men articulated a general mistrust of PrEP and TasP as reliable prevention methods. They described preferring to rely on condoms, or more commonly, selecting sexual partners on the basis of their (often assumed) HIV‐negative status. Underlying these strategies were concerns about homophobia and the role it could play in blurring distinctions between being at high risk of HIV and being HIV‐positive:

R1 Wi’ cancer … a lot o’ people … well they know definitely they can't catch cancer, but you've got so many old fashioned folk still in this world that if you … If your laundry was aired publicly that you had HIV, you'd lose friends like that [clicks fingers]. You'd walk along the high street an’ people would move out the way, d'you know what I mean? Because a lot of people are just so … non‐educated about the different types of like infection an’ diseases you can get, d'you know what I mean? I mean I can imagine me, if I had HIV an’ everybody knew like all these little school kids just like, “oh he's got AIDS, get away from him,” d'you know what I mean?

R2 ‘Cause you already get that even though you dinnae [don't] have it, you still get that folk that's homophobic sayin, “oh you've got AIDs, an’ you've got that”

R3 Because you're gay, like it's the stigma.

R2 …because you're gay.

(HIV‐negative/untested gay men)

These complexities of negotiating gay identities in a perceived homophobic society highlight how participants sought to resist the elision of being gay with being HIV‐positive. For these young men, limiting proximity to HIV could be seen as strategy to manage homophobia in the context of a society that was not ‘educated’ about HIV. Indeed, the men in this group repeatedly talked about maintaining confidentiality around their sexual health because they lived in a small community:

I couldn't go to [the local] GUM clinic ‘cause if I saw someone sittin’ ootside that I ken[knew], it would look like that I had everything, d'you know what I mean? Whereas my doctor they [don't know] what I'm goin’ in for an’ I find it a lot safer … So I go to my doctors I find it more secretive an’ confidential. (HIV‐negative/untested gay man)

While concerns about being seen at the local sexual health clinic are not limited to small communities, these anxieties highlight the work these men do in managing public perceptions of both their sexual identity and sexual health. This work has implications for HIV prevention; the intersections of different forms of stigma (i.e. homophobia, HIV‐stigma) and other social ‘risks’ (i.e. ignorance about HIV) could be managed in ways that distance or limit any engagement with HIV.

## The responsibility and culpability of HIV prevention

The biomedicalisation thesis posits that health has become a moral obligation. This confers upon the individual a responsibility to be and remain healthy, enforced through disciplinary forms of self‐governance (171–2). Similarly, discussions around the use of PrEP or TasP were framed by this sense of responsibility for HIV prevention and requirement of self‐governance. However, these discussions were also strongly shaped by the notion that certain groups of people – specifically people diagnosed with HIV – were more responsible than others for preventing transmission. This prevention imperative can be seen in how many HIV‐positive participants described managing their own health practices to reduce the risk of transmission to others. For instance, many HIV‐positive African women described how their testing practices related directly to preventing mother‐to‐child transmission. One woman, who learned her HIV status during her pre‐natal care, explained planning to test for HIV only when she became pregnant because of the transmission implications for the foetus, and not her own health. Before she was pregnant, she told herself: ‘you know what, no matter what, when I'm pregnant I'm going to test myself’ (HIV‐positive African woman). In relation to PrEP, the implications of this prevention imperative can be seen in anxieties expressed about trust and control by HIV‐positive participants. One woman explained her scepticism of PrEP as an effective prevention method because it was not her, but her sexual partner in control of it: ‘[PrEP] is too risky for him because I don't know when he stop using it, what will happen to him’ (HIV‐positive African woman). Instead of viewing her partner's potential non‐adherence to and subsequent diminished protection from PrEP as his responsibility, she took on the responsibility for both parties and dismissed PrEP as a potential option for her serodiscordant partnership.

This prevention imperative, and requisite forms of self‐governance, underpinned responses to PrEP and TasP. Although somewhat contested by HIV‐positive participants, there was a clear sense of the heightened social responsibility and culpability for managing the risks of HIV transmission. The following extract from a discussion with HIV‐positive gay men highlights how perceptions of responsibility for HIV prevention might affect potential uptake of PrEP or reliance on TasP:

R1 People with HIV have more responsibility.

R2 No.

R3 Yeah, I think …

R2 People are responsible for their own sexual health so I think both actually. Not necessarily someone using PREP because maybe the HIV‐negative person might, what the word I was looking for? … might resent starting PrEP ‘cause they're thinking ‘I'm starting this, taking these pills, they're making me feel rotten, why do I need to do this? Why can't I just use a condom?’ So maybe it's a bit of both.

(….)

R1 Generally I hear again and again, and it's sometimes directly or sometimes indirectly, men who are negative or presumed to be negative saying that if you're HIV‐positive you've got to protect everybody. It's your responsibility. I'm negative so it's nothing to do with me. So people who are HIV‐positive have all the responsibility to protect negative men. So in that scenario I wonder what they'd say ‘well obviously you start treatment, you're the one who's got the disease, I don't have it’.

R2 No I think it's up to …

R3 Everybody's responsible for their own …

R1 … not everybody like Peter, not everybody is encouraged to go on drugs straight away. So the negative person wouldnae [wouldn't] necessarily get the idea that although Peter is positive, he isnae [isn't] at risk yet.

R2 I think we're all responsible for our own actions.

(HIV‐Positive gay men)

Although there was some debate about whether responsibility for the prevention of HIV transmission was shared, or if HIV‐positive individuals were more responsible than others, this discussion also highlights the TasP‐specific anxieties of HIV‐positive participants in this regard: they may be held to account for either risk of HIV transmission, or the effects of taking medication to prevent HIV transmission. In other words, the stigma‐driven sero‐divide in responsibility exacerbates the culpability of HIV‐positive individuals when demanding additional behaviour/precautions by HIV‐negative sexual partners. This additional biomedical responsibility overlaps with a further element of biomedicalisation: the transformation of bodies to include new properties. In this case, we can see the increased responsibility of diagnosed HIV‐positive individuals to prevent transmission upon the acquisition of their new biomedical identity (Race 2001). However, participants felt that TasP complicated the social reception of this biomedical identity, in that not all diagnosed HIV‐positive people, like Peter, were on treatment. This raised concerns amongst participants that TasP could lead to a misinterpretation of the protection of ARVs as universal, further complicating their own role in preventing potential transmission.

While HIV‐positive participants were reassured by TasP in managing potential transmissions, there was a sense that this was in no way sufficient to offset the range of risks and responsibilities they incurred from being HIV‐positive. Moreover, the responsibility to manage HIV by this group was made apparent through the law:

R1 I think it's a good idea coz if you're less likely to pass it on, and that's a proven fact, I think that's a good idea – coz you're then preventing, you're reducing the risk to other people so you're then reducing other people who are on medication.

Q Mm, but you're increasing you're, the amount of time you're on medication.

R1 Does that really make any difference? Because if you're HIV‐positive and don't know the status of the person you're sleeping with, that you're more inclined to use condoms or position yourself differently so the risk is reduced to them. But it's also so the criminalisation of risk and the transmission risk as well, so is this really worth it? Because you can still get prosecuted for it, putting somebody at risk, even if it's just a nominal risk….

(HIV‐positive gay men)

Concerns about the potential prosecution of HIV transmission shaped the responses of some HIV‐positive participants to the possibility of using TasP; the role of the state and legal system in these concerns highlights how the institutional processes of biomedicalisation are present in the most intimate of contexts (Weait 2007).

## Conclusion

This article has explored the ways in which the acceptability and potential use of PrEP and TasP are affected by – and affect – processes of biomedicalisation. Yet, as we have seen, the biomedical does not function independently: the social, cultural and material shape the processes of biomedicalisation in varied and uneven ways. We found that responses to PrEP evoked the commodification of HIV prevention through the critiques of ‘Big Pharma’ and HIV science, as well as through implicit demands for more equitable health systems. Yet, not all were sceptical of the open market system in its ability to facilitate improved health options, including potential PrEP consumers. We also identified how deeply entrenched forms of HIV‐stigma and homophobia can shape and obfuscate the consumption of PrEP and TasP knowledge. At the same time, participants drew on certain forms of knowledge as liberating and as a way of overcoming stigma in the social management of PrEP. Finally, we found that responses to TasP and PrEP were shaped by the construction of HIV‐positive bodies as risky, and the accompanying responsibility of those with HIV‐diagnoses to manage this risk. Thus, rather than seeing TasP or PrEP as ‘liberating’, for instance through reduced levels of infectiousness or risk of transmission, social and legal requirements of responsibility in relation to HIV risk reinforced unequal forms of biomedical self‐governance.

Our findings echo and build on Persson's (2013) work, who found that biomedical messages about HIV infectiousness were met with ambivalence, grounded in contingent and changing experiences of HIV corporeality. Similarly, we found that the division between people diagnosed with HIV and those who are (or are assumed to be) HIV‐negative fundamentally shaped responses to TasP and PrEP, in spite of the potential of these prevention options to break down these very barriers. This has significant implications for not only the acceptability of these interventions, but also how PrEP and TasP are implicated in maintaining or exacerbating this biomedical stratification. Others have detailed the stratification of biomedicalisation in heart disease and how the disjuncture between expert and lay understandings were framed through race, gender and poverty (Clarke *et al*. 2010). Our work highlights how responses to TasP and PrEP are grounded not solely in an expert/lay divide or economic inequalities per se, but in a sero‐divide that is perpetuated through the ‘emergent dividing practices’ (Clarke *et al*. 2003: 184) of the very biomedical processes themselves. Yet, the uneven patterning of biomedicalisation is amplified and further manifested in the nexus of traditional lines of inequalities such as sexual identity, ethnicity, migration/mobility, gender and geography. Critiques of these biomedical prevention interventions are therefore right to be grounded in the language of biomedicalisation. Our work details how, in addition to overarching biomedicalised policy shifts (Kippax and Stephenson 2012), the subtle biomedicalised transformations at the micro level have significant implications for TasP and PrEP negotiations.

Our work was exploratory and suggestive of the heterogeneous effect of biomedicalisation on particular HIV interventions. However, our findings suggest a potentially growing gulf between HIV‐positive and HIV‐negative communities in terms of health expectations and experiences of negotiating biomedical prevention. It also raises a number of questions about what equitable and fair HIV prevention looks like, how this might manifest across diverse communities and who is or will be implicated in the increasingly biomedical moral imperative to prevent HIV. As Holt (2014) suggests, this has not been, nor will be a straightforward process. Yet as prevention technologies like TasP and PrEP increasingly become more regular facets of biomedicalised HIV practices, we need to attend to the material, lived and stratified experiences of diverse communities affected by HIV.

## Notes

1 For the sake of brevity, we will refer to this group as gay men.

2 We refer here to diverse communities as this group includes men and women from multiple, sub‐Saharan African countries where HIV is endemic. Again, for brevity, we will refer to this group as African men and women.
